# Longitudinal Outcomes of Psychiatric Inpatients After Suicide Attempt: A Retrospective Study in Portugal

**DOI:** 10.1192/j.eurpsy.2026.10218

**Published:** 2026-07-31

**Authors:** M. J. Cunha, M. Cândido, G. Canhoto, M. Ribeiro, J. Domingos, S. Neto, A. Velosa

**Affiliations:** 1 Psychiatry, Unidade Local de Saude Lisboa Ocidental; 2 NOVA Medical School - Faculdade de Ciências Médicas da Universidade Nova de Lisboa, Lisbon, Portugal

## Abstract

**Introduction:**

Suicide attempts (SA) are the strongest predictor of future suicide. Hospitalization after SA is a critical clinical decision, yet longitudinal outcomes of this population remain poorly described in Portugal. Understanding socio-demographic and clinical risk factors is essential to guide prevention strategies.

**Objectives:**

To longitudinally characterize psychiatric inpatients admitted after SA and evaluate recurrence, rehospitalizations, and mortality.

**Methods:**

We conducted a retrospective observational study including all patients admitted to the Acute Psychiatric Inpatient Unit of Unidade Local de Saúde Lisboa Ocidental between January 2022 and August 2025, following a documented SA in the previous month. Socio-demographic, clinical, index-SA, and follow-up variables were collected from electronic health records. Descriptive statistics were applied.

**Results:**

Ninety-three patients were included (53.8% female; mean age 44.9 ± 19.0 years). A majority were single (60%), 54.8% were unemployed or otherwise inactive, and only 43% had children (mean 0.7), indicating fragile social support. Nearly half (48.4%) had a prior SA, 71% presented suicidal ideation, and 46.2% reported substance misuse; 21.5% had a family history of suicidal behaviour. The index SA was impulsive in 52.7%. Methods included drug overdose (49.5%), self-cutting (11.8%), defenestration (6.5%), and hanging (5.4%). Median length of stay was 17.5 days (IQR 11–33). Discharge diagnoses were mainly depressive disorders (52.7%), bipolar disorder (11.8%), borderline personality disorder (7.5%), and schizophrenia/psychosis (8.6%). During follow-up, 15.1% presented new non-fatal SAs (15 events), 7.5% required rehospitalization (18 episodes), and 2 patients (2.2%) died by suicide. Three additional deaths of unknown cause were recorded.

**Conclusions:**

This is the first longitudinal characterization of SA-related psychiatric hospitalizations in Lisbon. Patients showed a profile of psychiatric morbidity combined with marked social vulnerability (isolation, unemployment, absence of children) and frequent substance misuse. Recurrence of suicidal behaviour and fatal outcomes occurred despite hospitalization, underscoring that inpatient care alone is insufficient. These findings highlight the need for multimodal post-discharge strategies integrating early follow-up, psychosocial and occupational support, and targeted interventions for substance misuse. Addressing social determinants alongside psychiatric treatment should be a cornerstone of suicide prevention.

**Disclosure of Interest:**

None Declared

